# Inefficacious drugs against covid-19: analysis of sales, tweets, and search engines

**DOI:** 10.11606/s1518-8787.2024058005413

**Published:** 2024-02-21

**Authors:** Irineu de Brito, Flaviane Azevedo Saraiva, Nathan de Campos Bruno, Roberto Fray da Silva, Celso Mitsuo Hino, Hugo Tsugunobu Yoshida Yoshizaki

**Affiliations:** I Universidade Estadual Paulista “Júlio de Mesquita Filho” Instituto de Ciência e Tecnologia Departamento de Engenharia Ambiental São José dos Campos SP Brasil Universidade Estadual Paulista “Júlio de Mesquita Filho”. Instituto de Ciência e Tecnologia. Departamento de Engenharia Ambiental. São José dos Campos, SP, Brasil; II Universidade de São Paulo Escola Politécnica Programa de Mestrado em Engenharia de Sistemas Logísticos São Paulo SP Brasil Universidade de São Paulo. Escola Politécnica. Programa de Mestrado em Engenharia de Sistemas Logísticos. São Paulo, SP, Brasil; III Universidade de São Paulo Escola Politécnica Departamento de Engenharia de Produção São Paulo SP Brasil Universidade de São Paulo. Escola Politécnica. Departamento de Engenharia de Produção. São Paulo, SP, Brasil; IV Universidade de São Paulo Instituto de Estudos Avançados São Paulo SP Brasil Universidade de São Paulo. Instituto de Estudos Avançados. São Paulo, SP, Brasil

**Keywords:** Social Media, Chloroquine, Ivermectin, Covid-19, Search Engine, Infodemic, Pandemic, SARS-CoV-2

## Abstract

**OBJECTIVE:**

Assess the correlation between the sales of two drugs with no proven efficacy against covid-19, ivermectin and chloroquine, and other relevant variables, such as Google^®^ searches, number of tweets related to these drugs, number of cases and deaths resulting from covid-19.

**METHODS:**

The methodology adopted in this study has four stages: data collection, data processing, exploratory data analysis, and correlation analysis. Spearman’s method was used to obtain cross-correlations between each pair of variables.

**RESULTS:**

The results show similar behaviors between variables. Peaks occurred in the same or near periods. The exploratory data analysis showed shortage of chloroquine in the period corresponding to the beginning of advertising for the application of these drugs against covid-19. Both drugs showed a high and statistically significant correlation with the other variables. Also, some of them showed a higher correlation with drug sales when we employed a one-month lag. In the case of chloroquine, this was observed for the number of deaths. In the case of ivermectin, this was observed for the number of tweets, cases, and deaths.

**CONCLUSIONS:**

The results contribute to decision making in crisis management by governments, industries, and stores. In times of crisis, as observed during the covid-19 pandemic, some variables can help sales forecasting, especially Google^®^ and tweets, which provide a real-time analysis of the situation. Monitoring social media platforms and search engines would allow the determination of drug use by the population and better prediction of potential peaks in the demand for these drugs.

## INTRODUCTION

The covid-19 pandemic was declared a public health crisis by the World Health Organization in early March 2020. The disease caused millions of deaths around the world^1^. In the first two years of the pandemic, social and conventional media, as well as official bodies, mentioned several potential treatments for the disease without proven evidence of efficacy^1,2^. This information generated confusion among the population and an artificial increase on the demand for various pharmaceutical products^3^ for the potential treatment of covid-19^2,4^.

Information shared on social media related to uncertainties and unavailability of drugs contributed to the development of panic buying behavior among consumers during this period^5^, who mostly bought grocery items and, in some cases, drugs for potential treatments, causing a shortage of these products^3,6^. Many drugs advertised on social media platforms, such as Twitter^®^, have no proven evidence of efficacy against covid-19^7^. The inefficacious drugs promoted by official bodies^2^ included chloroquine and hydroxychloroquine (collectively referred to as “chloroquine” in this study) and ivermectin.

At the beginning of the pandemic, the Federal Government of Brazil recommended and distributed these drugs^2^ as an early treatment, in the so-called “Covid Kit.” This action, based on speculations that these drugs could prevent or treat the disease, was adopted without scientific studies demonstrating their efficacy^8^. The use of these products can cause health problems, both due to their side effects and the shortage to patients with prescribed use, as the sudden increase in demand led to a shortage of these drugs^3^.

The study period was from January 2020 to December 2021, which includes moments before the pandemic. This study analyzed data from Brazil only. It aimed to answer the following questions: 1) Did the monthly retail sales volumes of ivermectin and chloroquine correlate with trends on Google^®^ search engine and the number of posts on Twitter^®^?; and 2) Did the monthly retail sales of these products correlate with the number of cases or deaths from covid-19?

Data about tweets, Google^®^ searches (through Google Trends), and number of cases and deaths in Brazil were obtained and processed, in addition to monthly sales (provided by IQVIA^®^) of chloroquine and ivermectin. In the subsequent stages of this study, exploratory and correlation analyses were conducted.

Similar statistical methods to those used in our study, including exploratory analysis, autocorrelation, and Spearman’s correlation, have been used in studies on drugs and social media to analyze correlations with sales and false and malicious information about the pandemic^2,3,9-11^.

Other studies have used data from Google Trends to evaluate sales of hydroxychloroquine and ivermectin^8^ in the United States and Canada, as well as chloroquine, remdesivir, paracetamol, and ibuprofen in Australia, Germany, Italy, Spain, the United Kingdom, and the United States^9^. Other studies used search engines in conjunction with e-commerce sales data to evaluate sales behavior after statements on Twitter by influential people abroad supporting the use of chloroquine^12,13^. Some studies^8,14,15^ used IQVIA^®^ data as a source of information on product sales during the pandemic. Studies about the pandemic in Brazil using a similar approach assessed the use of these drugs during the pandemic, their adverse effects^10^, and the impact on their prices^14^.

However, none of these studies considered the volume of drugs sold over the entire study period studied (two years). Also, most of them focused on just one drug, which resulted in a limited view of panic buying of drugs without proven evidence of efficacy against covid-19.

The main contribution of this study is that it provides information to support the decision-making process in crisis management by both government agents and drug supply chain managers by using tools that can anticipate sales or detect panic buying behavior induced by social media.

This article has six sections. The introduction describes the context, motivations, and objectives of this study. The second section briefly addresses the importance and use of drugs analyzed in this study. The third section describes the study methods. The results and discussions are presented in the fourth and fifth sections, respectively. The last section contains conclusions, study limitations, and suggestions for future studies.

### Chloroquine and ivermectin: importance and use

Chloroquine is an anti-inflammatory drugs used to treat various conditions, including malaria, rheumatoid arthritis, and lupus erythematosus^2^. Chloroquine and hydroxychloroquine are similar drugs. The main difference between them is the slightly modified molecular structure of hydroxychloroquine, which can result in improved efficacy and fewer side effects, allowing its use in smaller doses and longer periods when compared to chloroquine^16^.

Ivermectin is an antiparasitic drug used to treat infections caused by worms and other parasites^17^, such as scabies and onchocerciasis^18^.

## METHODS

The study method has four stages: 1) data collection; 2) data processing; 3) exploratory analysis; and 4) correlation analyses between data. Data about chloroquine and ivermectin were processed and analyzed separately. The following Python packages were used in this study: TWINT, NumPy, Pandas, Matplotlib, SciPy, Seaborn, Statsmodels, and Scikit-learn.

In the first stage, data were collected from January 1, 2020 to December 31, 2021 from four sources, as described below:

1. Number of tweets in Portuguese, containing keywords related to the active ingredient and commercial names related to chloroquine (hydroxychloroquine, hidroxicloroquina, chloroquine*,* cloroquina, diclokin, quinacris, plaquinol, and reuquinol), and ivermectin (ivermectin*,* ivermectina, revectina, iverlat, and vermectil);
2. Number of searches per week in Brazil on Google^®^, using the Google Trends platform and the terms chloroquine and ivermectin. This platform shows the evolution of terms searched on Google^®^, according to the location and period specified by the user^8^. Search tools, such as Google^®^, were used by the population to obtain information related to potential drugs and treatments for covid-19;
3. Daily cases and deaths of covid-19in Brazil, based on the database provided by the University of Oxford^19^;
4. Monthly retail sales of chloroquine and ivermectin in Brazil according to IQVIA^®^, a leading global provider of advanced analytics and clinical research^20^.

In the second stage, all variables were grouped by month because the sales volume was monthly. Due to sales data confidentiality, all data were normalized according to the MinMax standardization, in which the minimum value of each series is transformed into 0 and the maximum value is transformed into 1.

In the third stage, exploratory data analyses were conducted. The time series were plotted on graphs for visual analysis of the relationship between the sales volume of each drug and the other variables. The autocorrelation function (ACF) and partial autocorrelation function (PACF) were applied to explore the relationship between previous and subsequent values of the dataset over time.

ACF measures the correlation between past and future values in a time series, showing the degree of linear dependence of data, while PACF measures the direct correlation between an observation and its previous values, without the influence of intermediate observations. In the case of time series of monthly sales of drugs, the use of ACF and PACF identifies whether sales: 1) are influenced by previous months; and 2), if so, whether they are influenced by several previous months (ACF) or only by the previous month (PACF).

These analyses were conducted using correlograms^21^, which display significant correlations in the data series to identify the existence of serial dependence. Although the dataset is small (24 months), these analyses were essential for the identification of potential trends in the data.

In the last stage, cross-correlation analyses were performed. Using the Shapiro-Wilk^22^ test, data normality was analyzed. Scatter plots were used for linearity analysis. Based on these results, Spearman’s correlation was used to measure the correlation between the variables. Cross-correlation^10^ was then applied to analyze the relationship between the sales of each drug, assuming time lags of zero to three months for each of the variables analyzed in relation to the sales volume.

## RESULTS

This section contains the results obtained after applying the methods explained above. For a better interpretation, the results are presented separately for chloroquine and ivermectin.

### Chloroquine

**Figure 1:**
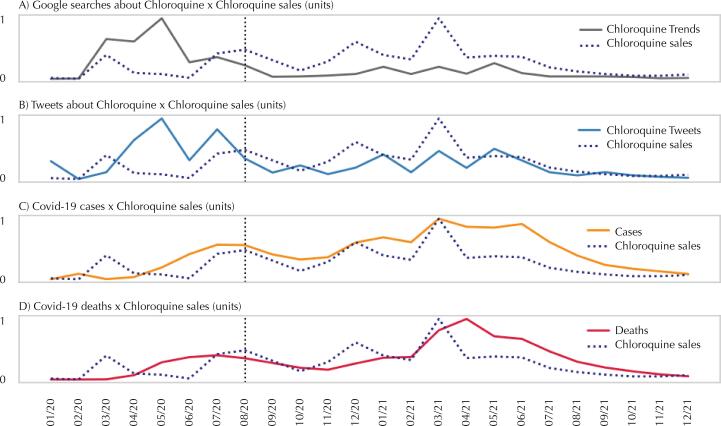
Chloroquine sales, Google® searches, number of related tweets, cases and deaths (normalized data).

Figure 1 shows the different time series analyzed in relation to the volume of chloroquine sales. The peak sales (dashed line) occurred in March 2021, as well as the peak in number of cases (Figure 1c) and deaths (Figure 1d) due to covid-19. On the other hand, Google^®^ searches (Figure 1a) and the number of tweets (Figure 1b) showed peaks in May 2020, still in the initial phase of the pandemic, indicating a similar behavior to panic buying^6^. This was mainly related to the euphoria of some world leaders, and chloroquine was introduced as an easy solution to the health crisis^23^. However, as noted above, there was no scientific evidence of its efficacy.

At the beginning of the pandemic, there was a global shortage of chloroquine^14,24^, which is illustrated in Figure 1, between March and August 2020 the vertical line at 08/20 identifies the end of the period). According to scientific articles, media news, and information obtained from drug manufacturers in Brazil and in countries such as the United States, Canada, the United Kingdom, Australia, India, and Pakistan, these shortages occurred until July 2020^25-28^. After that, manufacturers were able to fulfill the orders for the retail market. Therefore, due to the occurrence of these shortages, the exploratory and correlation analyses considered the period from August 1, 2020 to December 31, 2021.

The ACF and PACF analyses demonstrated an autocorrelation between the one-month lag and the series of the number of cases and deaths. The series of sales, Google^®^ searches, and number of tweets for chloroquine did not show autocorrelation.

Next, the Shapiro-Wilk test was applied to evaluate the assumption of data normality. The results showed that only the case series had a normal distribution (95% confidence level). The other series did not fulfill this assumption. According to the scatter plots between pairs of data series, the linearity condition was not met. For these reasons, the Spearman’s test was selected to analyze the correlations between the variables in the next stage.

Cross-correlation was used to investigate the relationship between chloroquine sales and other variables, assuming different time lags. All correlations were positive. The results indicated that, in some cases, the value of the correlation with lags in the sales variable was higher than that between the original series.

Table 1 shows that the best correlation (0.7903) between chloroquine sales and deaths (right column) occurs when considering a one-month lag at a 99.9% confidence level. In other words, a stronger correlation was observed when comparing deaths to chloroquine sales in the previous month than in the same month.

**Table 1:** Spearman’s correlation between each variable and chloroquine sales considering zero to three-time lags.

Sales lags (months)	Google^®^ searches	Tweets	Cases	Deaths
0	0.8833*	0.8167*	0.8179*	0.7002**
1	0.6325**	0.5784*	0.7932*	0.7903*
2	0.5722***	0.5607***	0.6250***	0.6821**
3	0.4558	0.4286	0.6264***	0.6088***

Correlation meanings: *0.001; **0.01; ***0.05 (bivariate).

For the variables of Google^®^ searches, number of tweets, and cases, the best correlation with chloroquine sales occurred between the original series without lags (Table 1). Google^®^ search variable showed the highest correlation with chloroquine sales (0.8833), but the numbers of tweets, cases, and deaths also showed positive and statistically significant correlations (99% confidence level).

### Ivermectin

**Figure 2:**
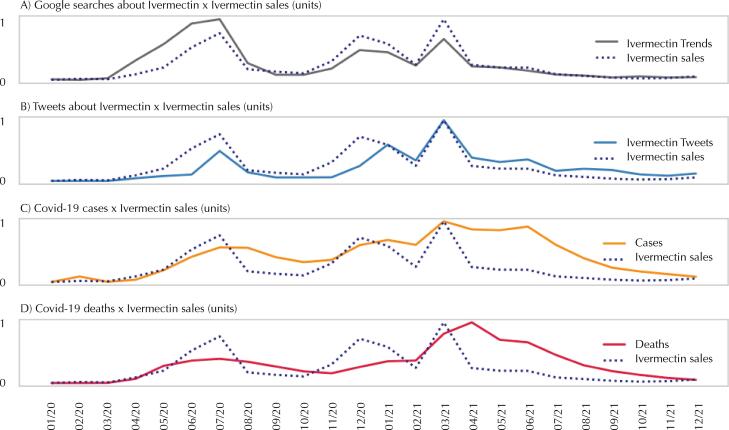
Ivermectin sales, Google® searches, number of related tweets, cases and deaths (normalized data).

Figure 2 shows the series of sales, number of tweets, and Google^®^ searches related to ivermectin, cases, and deaths. As in the case of chloroquine, the peak in ivermectin sales was observed in March 2021, coinciding with the peak number of tweets related to the drug. Regarding Google^®^ search results, the peak was in July 2020.

In the case of ivermectin, no news or scientific articles were identified reporting a shortage in the retail market. It was also not observed in the sales data.

Then, the autocorrelation of the ivermectin-related series was assessed. The series of Google^®^ searches and the number of tweets presented autocorrelation with a one-month lag. Ivermectin sales, however, showed a significant lag only in direct autocorrelation (PACF) with a one-month lag.

The variables of number of tweets and Google^®^ searches related to ivermectin did not present a normal distribution during the study period – no linear relationship with sales, as assessed using the Shapiro-Wilk test and scatter plots, respectively.

Table 2 shows the results of the cross-correlation between the variables, considering time lags of zero to three months. Stronger correlations were observed for the number of tweets, cases, and deaths with a one-month lag in the sales variable (99% confidence level).

**Table 2:** Spearman’s correlation between each variable and ivermectin sales considering zero to three-time lags.

Sales lags (months)	Google^®^ searches	Tweets	Cases	Deaths
0	0.9100*	0.6626*	0.7461*	0.7054*
1	0.6822*	0.7490*	0.8607*	0.8202*
2	0.3068	0.5968**	0.8227*	0.7549*
3	-0.1436	0.3753	0.6649**	0.5675**

Correlation meanings: *0.001; **0.01; ***0.05 (bivariate).

Only the variable of Google^®^ searches showed a higher correlation (0.9100) when considering the original series of ivermectin sales. As seen with chloroquine, the variable with the highest correlation with ivermectin sales was Google^®^ searches.

Table 2 shows the relationship between the variables and does not guarantee the existence of causality. Several factors may have caused time lag, including the delay in reporting cases and deaths, as indicated in Brazilian news and studies on disease monitoring^29^.

## DISCUSSION

A significant positive correlation (at least 95% confidence) was observed between sales and all variables analyzed (Google^®^ searches, number of tweets, cases, and deaths), lags of zero or one month (Tables 1 and 2), and both drugs. In particular, Google^®^ searches consistently demonstrated the highest correlation with sales, always with zero lag, for both chloroquine and ivermectin, with correlations of 0.8833, 0.9100, and 99.9% confidence. Therefore, this variable seems to be an excellent indicator for sales variations.

The number of tweets also presented a high positive correlation with sales, but the highest correlations were observed at different moments for chloroquine and ivermectin: zero lag (0.8167) and one-month lag (0.7469), respectively, both with 99.9% confidence level. For ivermectin, the correlation with sales for zero lag is relatively high (0.6626) and significant (99.9%). Therefore, the number of tweets is a good indicator of sales variation and can be used with Google^®^ searches. However, the different lags (between the two drugs) for the highest correlation suggest the number of tweets should be considered more carefully.

For the variable of the number of deaths, the best correlation for both drugs has been observed with a one-month lag (0.7903 and 0.8202) at 99.9% confidence level. For the variable of the number of cases, similarly to the number of tweets, the best correlation for chloroquine was observed with no lag (0.8179), while for ivermectin, it was observed with a one-month lag (0.8607), both at 99.9% confidence level.

The fact that all correlations are positive seems to support a relation between the variables and sales; that is, when any variable grows, sales also increase.

From a drug supply chain perspective, the variables of Google^®^ search and the number of tweets provide a real-time assessment of the situation that helps anticipate demand and speed up the decision-making process^30^ in the epidemiological management of the disease. The variables traditionally used for this purpose (numbers of cases and deaths) are reported after their occurrence^31^, not allowing their proactive use.

Google Trends provides the number of searches on Google^®^ and it is easy to use – the user simply types the search term, the desired period, and the region or location of interest). However, specific computer programs are required to obtain the number of tweets.

Thus, monitoring Google^®^ searches and the number of tweets would allow estimating potential peaks in the demand for inefficacious drugs in a future pandemic, which would allow actions to avoid shortages for users who actually need these drugs, as occurred with chloroquine. Such actions could be the temporary requirement of medical prescriptions, removal of the drug from internet sales platforms, or limitation of the amount sold per customer.

Academic publications reported the behavior of famous people who, through social or conventional media, promoted the use of inefficacious drugs against covid-19^13^, causing an increase in the search for chloroquine in the United States when compared to other countries in Europe and Oceania^9^, and Canada^8^. It increased the sales of these products locally^12^. In Brazil, constant statements and posts by the president about drugs generated misinformation and boosted search for such medations^1,32^.

As a suggested process, public health agents could adopt the following measures in similar pandemic situations: (1) identify important people to disseminate true information on conventional and social media; (2) monitor the number of messages related to the topic on social media, considering both the total number of posts and the posts by the important people identified in item 1; (3) monitor the number of searches related to the topic conducted in relevant search tools; and (4) use the methods applied in this study (Spearman’s correlation, cross-correlation with multiple lags, exploratory analysis, and autocorrelation) to compare the evolution of the disease with other time series (such as drug sales, cases, deaths, number of searches, and number of messages).

These measures would help identify: (1) a potential increase in demand in real-time; (2) potential panic buying behavior, allowing better supply of the product by the supply chain; and (3) new trends regarding demands for the drugs in question. Together, these aspects can support an adequate planning and adoption of strategies by supply chain management agents to mitigate a shortage of drugs, which is essential for patients who actually need these drugs.

## CONCLUSIONS

Chloroquine and ivermectin were two drugs indicated at the beginning of the pandemic for the treatment of covid-19, although there were no scientific studies that proved their efficacy. Due to the influence of social media, conventional media, and government agents^2,4^, many people from different countries around the world used these drugs.

This article analyzed the existence of a correlation between the volume of monthly retail sales of these drugs in Brazil with the following variables: Google^®^ searches, the number of tweets related to these drugs, and the numbers of cases and deaths by covid-19 during the first two years of the pandemic in Brazil.

The main contribution of this study refers to the high correlation of the variables analyzed. In times of crisis, as observed during the pandemic, they were able to assist sales forecasting, in particular Google^®^ and tweets, which provide a real-time analysis of the situation. For other variables (cases and deaths), this correlation was stronger, when considering a time lag in the sales variable, which makes them less adequate for a forecasting study.

These results also point to a stronger need for the development of crisis management actions on social media, due to its relationship with consumer behavior. Monitoring social media platforms and the evolution of searches on search engines provides a better prediction of potential peaks in demand, allowing better supply planning by supply chain agents. The importance of considering the impact of traditional and social media on consumer behavior, especially in times of crisis, is highlighted in this study.

The main study limitations include: (1) the study period is restricted to 24 months, not allowing seasonality analysis over a longer period; and (2) the fact that the content and sentiment of tweets were not analyzed. Data generated contain tweets that are both for and against the use of these drugs. Therefore, future studies should consider sentiment and content analysis of tweets to improve sales forecasting models.
