# Supplementary material for: Inefficacious drugs against covid-19: analysis of sales, tweets, and search engines
Source: Rev Saude Publica. 2024 Feb 21;58:06. doi: 10.11606/s1518-8787.2024058005413 (PMC10926985; doi:10.11606/s1518-8787.2024058005413)
Supplement: Translation

# Translation: Medicamentos ineficazes contra covid-19: análise de vendas, tweets e mecanismos de busca

**Authors:** Irineu de Brito, Flaviane Azevedo Saraiva, Nathan de Campos Bruno, Roberto Fray da Silva, Celso Mitsuo Hino, Hugo Tsugunobu Yoshida Yoshizaki

**Affiliations:** I Brasil Universidade Estadual Paulista “Júlio de Mesquita Filho”. Instituto de Ciência e Tecnologia. Departamento de Engenharia Ambiental. São José dos Campos, SP, Brasil; II Brasil Universidade de São Paulo. Escola Politécnica. Programa de Mestrado em Engenharia de Sistemas Logísticos. São Paulo, SP, Brasil; III Brasil Universidade de São Paulo. Escola Politécnica. Departamento de Engenharia de Produção. São Paulo, SP, Brasil; IV Brasil Universidade de São Paulo. Instituto de Estudos Avançados. São Paulo, SP, Brasil

**Keywords:** Mídias Sociais, Cloroquina, Ivermectina, Covid-19, Ferramenta de Busca, Infodemia, Pandemia, SARS-CoV-2

## Abstract

**OBJETIVO:**

Investigar a correlação entre as vendas de dois medicamentos sem eficácia comprovada no tratamento de covid-19, ivermectina e cloroquina, e outras variáveis relevantes: pesquisas no Google®, número de *tweets* relacionados aos medicamentos, casos e óbitos decorrentes da covid-19.

**MÉTODOS:**

A metodologia adotada neste estudo se divide em quatro partes: coleta de dados; processamento dos dados; análise exploratória; e análise de correlação. Foi utilizado o método de Spearman para obter as correlações cruzadas entre cada par de variáveis.

**RESULTADOS:**

Os resultados mostram similaridade entre os comportamentos das variáveis. Os picos ocorreram em períodos iguais ou próximos. A análise exploratória dos dados apontou que houve falta de cloroquina no período correspondente ao início das divulgações sobre a aplicação desses medicamentos para o tratamento da covid-19. Ambos os medicamentos apresentaram correlação alta e estatisticamente significativa com as demais variáveis analisadas. Também foi observado que algumas delas apresentaram maior correlação com as vendas de medicamentos quando assumiram defasagem temporal de um mês. No caso da cloroquina, isso ocorreu com a variável óbitos. No caso da ivermectina, ocorreu com as variáveis número de *tweets*, casos e óbitos.

**CONCLUSÕES:**

Os resultados observados contribuem para a tomada de decisão durante a gestão de crises por parte de governo, indústrias e comércios. Em momentos de crises, como observado durante a pandemia, as variáveis mostraram que são capazes de auxiliar na previsão de vendas, em especial o Google® e os *tweets,* que proporcionam uma análise em tempo real da situação. Acompanhar as redes sociais e mecanismos de busca permitiria detecção de uso pela população e melhor previsão de potenciais picos de demanda desses medicamentos.

## INTRODUÇÃO

A pandemia de covid-19 foi uma crise de saúde pública oficializada pela Organização Mundial da Saúde no início de março de 2020. A doença foi responsável por milhões de óbitos em todo o mundo^1^. Nos dois primeiros anos da pandemia, mídias sociais e convencionais, bem como organismos oficiais citaram diversos tratamentos potenciais para a doença sem garantia de eficácia cientificamente comprovada^1,2^. O compartilhamento desse tipo de informação gerou confusão na população e um aumento artificial na demanda de vários produtos farmacêuticos^3^ para o potencial tratamento da covid-19^2,4^.

O compartilhamento de informações em mídias sociais relacionadas a incertezas e à indisponibilidade de produtos contribuiu para o desenvolvimento do comportamento de compras em pânico nos consumidores do período^5^. Estudos constataram a ocorrência dessas compras em itens de mercearia^4,7^ e, em alguns casos, em medicamentos relacionados a esses tratamentos, ocasionando a falta deles^3,6^. Muitos dos medicamentos divulgados em redes sociais, como o *Twitter*®, não possuem eficácia cientificamente comprovada para tratamento da covid-19^7^. Entre os medicamentos ineficazes promovidos por órgãos oficiais^2^, destacam-se a cloroquina e a hidroxicloroquina (avaliados conjuntamente neste trabalho sob o termo “cloroquina”) e a ivermectina.

No início da pandemia, o Governo Federal do Brasil recomendou e distribuiu esses medicamentos^2^ como forma de tratamento precoce, no chamado “Kit covid”. Essa ação, baseada em especulações de que as medicações poderiam prevenir ou tratar precocemente a doença, foi adotada sem estudos científicos rigorosos que comprovassem sua eficácia^8^. O uso desses produtos pode gerar problemas de saúde, tanto por seus efeitos colaterais quanto pelo desabastecimento aos pacientes com uso prescrito, pois o aumento súbito da demanda gerou a falta desses fármacos^3^.

O período analisado foi de janeiro de 2020 até dezembro de 2021, o que inclui momentos anteriores à pandemia. Foram utilizados dados referentes ao Brasil. O objetivo deste trabalho é responder às seguintes perguntas de pesquisa: 1) Os volumes mensais de vendas no varejo de ivermectina e de cloroquina correlacionaram-se com as tendências de pesquisas na plataforma Google® e com o número de mensagens na rede social *Twitter*®?; e 2) os volumes mensais de vendas no varejo desses produtos correlacionaram-se com casos e/ou óbitos por covid-19?

Foram obtidos e tratados dados sobre *tweets*, buscas no Google® (por meio da ferramenta *Google Trends*), casos e óbitos no Brasil, além de as vendas mensais (providos pela IQVIA®) de cloroquina e ivermectina. Nas etapas posteriores do trabalho foram realizadas análises exploratórias e de correlação.

Métodos estatísticos similares ao nosso trabalho, englobando análises exploratórias, autocorrelação e correlação de Spearman, aparecem em estudos sobre medicamentos e redes sociais para analisar as correlações com vendas e informações falsas e mal-intencionadas sobre a pandemia^2,3,9^.

Outros trabalhos utilizaram dados do *Google Trends* para avaliar vendas de hidroxicloroquina e ivermectina^8^ nos EUA e Canadá, assim como de cloroquina, remdesivir, paracetamol e ibuprofeno na Australia, Alemanha, Itália, Espanha, Reino Unido e EUA ^9^. Outros estudos empregaram mecanismos de busca em conjunto, com dados de vendas de *e-commerce* para avaliar o comportamento de vendas após declarações favoráveis, no *Twitter,* ao uso de cloroquina por pessoas influentes no exterior^12,13^. Alguns trabalhos^8,14,15^ utilizaram dados do IQVIA® como fonte de informação sobre vendas de produtos durante a pandemia. Estudos sobre o Brasil, com abordagem similar, foram publicados avaliando os impactos da utilização desses produtos durante a pandemia, analisando os efeitos adversos^10^ e o impacto nos preços desses medicamentos^14^.

No entanto, nenhum daqueles trabalhos considerou as vendas ao longo de todo o período estudado (dois anos). Ademais, a maioria deles focou somente um produto, o que resultou em uma visão limitada de compras em pânico dos medicamentos sem eficácia comprovada para o tratamento de covid-19.

A principal contribuição deste trabalho é fornecer informações que auxiliem o processo de tomada de decisão durante a gestão de crises tanto por parte de agentes de governo como de gestores de cadeias de suprimentos de medicamentos, ao demonstrar ferramentas capazes de antecipar vendas ou detectar comportamento de compras em pânico por indução das mídias sociais.

Este texto está organizado em seis seções. A introdução contextualiza e descreve as motivações e objetivos da pesquisa. A segunda seção aborda, sucintamente, a importância e o uso dos medicamentos que compõem o objeto do estudo. A terceira descreve a metodologia utilizada. Os resultados e discussões são apresentados na quarta e na quinta, respectivamente. A última seção contém conclusões, limitações e recomendações para pesquisas futuras.

### Cloroquina e ivermectina: importância e uso

A cloroquina é um medicamento antimalárico e anti-inflamatório utilizado em diversas condições, incluindo malária, artrite reumatoide e lúpus eritematoso^2^. A cloroquina e a hidroxicloroquina são medicamentos similares. A principal diferença entre os dois é a estrutura molecular ligeiramente modificada apresentada pela hidroxicloroquina, o que pode resultar em maior eficácia e menores efeitos colaterais, além de ser usada em doses menores e por períodos mais longos do que a cloroquina^16^.

A ivermectina é um medicamento antiparasitário utilizado para tratar infecções por vermes e outros parasitas^17^, como sarna, escabiose e oncocercose^18^.

## MÉTODOS

A metodologia tem quatro etapas: 1) coleta de dados; 2) processamento de dados; 3) análise exploratória; e 4) análises de correlação entre os dados. Os dados de cloroquina e ivermectina foram processados e analisados separadamente. Os seguintes pacotes em *Python* foram utilizados neste trabalho: *TWINT, NumPy, Pandas, Matplotlib, SciPy, Seaborn, Statsmodels* e *Scikit-learn*.

Na primeira etapa, dados de quatro fontes foram coletados para o período de 01/01/2020 a 31/12/2021:

1. Número de *tweets,* em português, contendo palavras-chave relativas ao princípio ativo e nomes comerciais relacionados à cloroquina (*hydroxychloroquine*, hidroxicloroquina, cloroquina, *chloroquine, diclokin, quinacris, plaquinol e reuquinol*) e à ivermectina (ivermectina, *ivermectin*, revectina, iverlat e vermectil);
2. Número de pesquisas no Google®, utilizando a plataforma *Google Trends,* com os termos “cloroquina” e “ivermectina” no Brasil, por semana. Essa plataforma mostra a evolução de termos pesquisados no Google®, conforme a localização e o período especificados pelo usuário^8^. Ferramentas de pesquisa, como o Google®, foram utilizadas pela população para obter informações relacionadas aos potenciais medicamentos e tratamentos para covid-19;
3. Casos e óbitos diários de covid-19 no Brasil, a partir da base de dados disponibilizada pela Universidade de Oxford^19^;
4. Número de vendas de cloroquina e ivermectina (mensalmente) no varejo no Brasil, fornecidos pela IQVIA®, provedora de dados e análises avançadas em saúde e pesquisa clínica^20^.

Na segunda etapa, todas as variáveis foram agrupadas mensalmente, visto que as variáveis de vendas foram disponibilizadas em intervalos mensais. Em função da confidencialidade dos dados de vendas, todos os dados foram normalizados seguindo a padronização *MinMax*, na qual o menor valor de cada série é igualado a 0, e o maior igualado a 1.

Na terceira etapa, foram conduzidas análises exploratórias dos dados. As séries temporais foram plotadas em gráficos para a análise visual da relação entre o volume de vendas de cada medicamento e as demais variáveis. As análises de autocorrelação (ACF – do inglês, *autocorrelation function*) e a autocorrelação parcial (PACF – do inglês, *partial autocorrelation function*) foram aplicadas, para explorar a relação entre valores anteriores e posteriores do conjunto de dados ao longo do tempo.

A ACF mede a correlação entre valores passados e futuros em uma série temporal, mostrando o grau de dependência linear dos dados, enquanto a PACF mede a correlação direta entre uma observação e seus valores anteriores, isolando a influência das observações intermediárias. No caso da série temporal de volume mensal de vendas dos medicamentos, o uso de ACF e PACF permite identificar se as vendas: 1) são influenciadas pelos meses anteriores; e 2), caso positivo, se são influenciadas por vários meses anteriores (ACF) ou apenas pelo mês imediatamente anterior (PACF).

Essas análises foram realizadas com correlogramas^21^, que permitem visualizar correlações significativas na série de dados, para identificar a existência de dependência serial. Embora o conjunto de dados seja pequeno (24 meses), essas análises foram essenciais para identificar potenciais tendências.

A última etapa executou análises de correlação cruzada. Por meio do teste de Shapiro-Wilk^22^, a normalidade dos dados foi verificada. Gráficos de dispersão foram utilizados para a análise de linearidade. Com base nesses resultados, optou-se pelo uso da correlação de Spearman como método mais adequado para a medição da correlação entre as variáveis estudadas. A correlação cruzada^10^ foi, então, aplicada para analisar a relação entre as vendas de cada medicamento, assumindo defasagens temporais (ou *lags*) de zero a três meses para cada uma das variáveis analisadas em relação ao número de vendas dos medicamentos.

## RESULTADOS

Nesta seção, observa-se a apresentação dos resultados obtidos pela aplicação dos métodos explicados anteriormente. Para melhor interpretação, foi dividida em duas partes: cloroquina e ivermectina.

### Cloroquina

A Figura 1 apresenta as diferentes séries temporais analisadas em relação ao número de vendas de cloroquina. Observa-se que o pico de vendas (série tracejada) ocorreu em março de 2021, assim como o pico de casos (Figura 1c) e de óbitos (Figura 1d). Por outro lado, as pesquisas no Google® (Figura 1a) e o número de *tweets* (Figura 1b) apresentaram picos em maio de 2020, ainda na fase inicial da pandemia, indicando comportamento similar ao de compras em pânico^6^. Isso está relacionado, principalmente, à euforia de alguns líderes mundiais e a apresentação da cloroquina como uma solução fácil para a crise sanitária^23^. No entanto, como observado anteriormente, não existiam comprovações científicas de sua eficácia.

**Figura 1:** Vendas de cloroquina, pesquisas no Google®, número de *tweets*, casos e óbitos relacionados (dados normalizados).

No início da pandemia houve falta de cloroquina globalmente^14,24^. Isso pode ser observado na Figura 1, no período entre março e agosto de 2020 (linha horizontal em 08/20 identifica o final do período). Segundo artigos científicos, notícias da mídia e informações obtidas junto aos fabricantes de medicamentos no Brasil e em países como EUA, Canadá, Reino Unido, Austrália, Índia e Paquistão, essas faltas ocorreram até julho de 2020^25^. A partir desse mês, os fabricantes conseguiram suprir os pedidos do mercado varejista. Assim, em função da ocorrência dessas faltas, a análise exploratória e a análise de correlação consideraram o período de 01/08/2020 a 31/12/2021.

As análises de ACF e PACF foram realizadas e demonstraram a existência de autocorrelação da defasagem de um período com as séries de casos e óbitos. As séries de vendas, pesquisas no Google® e número de *tweets* sobre cloroquina não apresentaram autocorrelação.

Na sequência, o teste de Shapiro-Wilk foi aplicado para a verificação da suposição de normalidade dos dados. Os resultados mostraram que apenas a série de casos segue uma distribuição normal (com 95% de nível de confiança). As demais séries não atenderam a essa suposição. Os gráficos de dispersão entre os pares de séries de dados indicaram que a condição de linearidade não é atendida. Por esses motivos, optou-se pelo teste de Spearman para analisar as correlações entre as variáveis na etapa seguinte.

A correlação cruzada foi utilizada para investigar a relação entre as vendas de cloroquina e as demais variáveis, assumindo diferentes defasagens temporais. Todas as correlações são positivas. Os resultados indicaram que, em alguns casos, o valor da correlação com defasagens na variável vendas foi maior do que entre as séries originais.

A Tabela 1 mostra que a melhor correlação (0,7903) entre vendas de cloroquina e óbitos (coluna da direita) ocorre quando se considera uma defasagem de um mês, com confiança de 99,9%. Em outras palavras, há uma correlação maior quando se compara os óbitos com as vendas de cloroquina do mês anterior, do que com o mesmo mês.

**Tabela 1:** Correlação de Spearman entre cada variável e as vendas de cloroquina considerando de zero até três defasagens temporais.

Defasagens vendas (meses)	Pesquisas no Google®	Tweets	Casos	Óbitos
0	0,8833 *	0,8167 *	0,8179 *	0,7002 **
1	0,6325 **	0,5784 *	0,7932 *	0,7903 *
2	0,5722 ***	0,5607 ***	0,6250 ***	0,6821 **
3	0,4558	0,4286	0,6264 ***	0,6088 ***

Significado da correlação: * 0,001; ** 0,01; *** 0,05 (bicaudal).

Para as variáveis de resultados de pesquisas no Google®, número de *tweets* e casos, a maior correlação com vendas de cloroquina ocorreu entre as séries originais, sem defasagens (Tabela 1). A variável de pesquisas no Google® apresentou a maior correlação com as vendas de cloroquina (0,8833), mas número de *tweets*, casos e óbitos também apresentaram correlações positivas e estatisticamente significativas (nível de 99%).

### Ivermectina

A Figura 2 apresenta as séries de vendas, número de *tweets* e de pesquisas no Google® relacionados à ivermectina, casos e óbitos. Assim como no caso da cloroquina, o pico de vendas de ivermectina ocorreu em março de 2021, coincidindo com o pico de número de *tweets* relacionados ao medicamento. Em relação ao resultado de pesquisas no Google®, o pico foi registrado em julho de 2020.

**Figura 2:** Vendas de ivermectina, pesquisas no Google® e número de *tweets*, casos e óbitos relacionados (dados normalizados).

No caso de ivermectina, não foram identificadas notícias ou artigos científicos que relataram sua falta no mercado varejista. Isso também não foi observado nos dados de vendas.

Em seguida, a autocorrelação das séries relacionadas à ivermectina foram exploradas. Observou-se que as séries de pesquisas no Google® e número de *tweets* apresentam autocorrelação com defasagem de um período. As vendas de ivermectina, no entanto, apresentaram defasagem significativa apenas quanto à autocorrelação direta (PACF), com defasagem de um período.

As variáveis número de *tweets* e pesquisas no Google® relacionadas à ivermectina também não seguiram distribuição normal durante o período analisado: não apresentaram relação linear com as vendas, conforme avaliou-se por meio do teste de Shapiro-Wilk e dos gráficos de dispersão, respectivamente.

A Tabela 2 apresenta os resultados da correlação cruzada entre as variáveis, considerando defasagens temporais de zero a três períodos (meses). As maiores correlações ocorrem para número de *tweets*, casos e óbitos quando temos a defasagem de um período na variável vendas, com nível de confiança de 99,9%.

**Tabela 2:** Correlação de Spearman entre cada variável e as vendas de ivermectina considerando de zero até três defasagens temporais.

Defasagens vendas (meses)	Pesquisas no Google®	Tweets	Casos	Óbitos
0	0,9100 *	0,6626 *	0,7461 *	0,7054 *
1	0,6822 *	0,7490 *	0,8607 *	0,8202 *
2	0,3068	0,5968 **	0,8227 *	0,7549 *
3	-0,1436	0,3753	0,6649 **	0,5675 **

Significado da correlação: * 0,001; ** 0,01; *** 0,05 (bicaudal);

Apenas a variável pesquisas no Google® apresentou maior correlação (0,9100) ao se considerar a série original de vendas de ivermectina. Assim como ocorreu com a cloroquina, a variável com maior correlação com as vendas de ivermectina foi pesquisas no Google®.

As correlações na Tabela 2 indicam a relação entre as variáveis e não garantem a existência de causalidade. A defasagem temporal pode ser resultado de diversos fatores. Uma possibilidade é o atraso da notificação de casos e óbitos, como relatado por notícias e pesquisas brasileiras sobre o monitoramento da doença^29^.

## DISCUSSÃO

Houve correlação positiva significativa (pelo menos 95% de confiança) de vendas para todas as variáveis analisadas (pesquisas no Google®, *tweets*, casos e óbitos), defasagens de zero ou um mês (Tabelas 1 e 2) e ambos os medicamentos. Em particular, a variável pesquisas no Google® demonstrou, consistentemente, ter a maior correlação com vendas, sempre com defasagem nula, tanto para cloroquina como para ivermectina, com correlações de 0,8833 e 0,9100 e confiança de 99,9%. Assim, essa variável parece ser um excelente indicador para variações de vendas.

A variável número de *tweets* também apresenta elevada correlação positiva com venda, mas as maiores correlações ocorrem em momentos separados para cloroquina e ivermectina: defasagem zero (0,8167) e de um mês (0,7469), respectivamente, ambos com confiança de 99,9%. Deve-se comentar que, para a ivermectina, a correlação com vendas para defasagem nula é relativamente alta (0,6626) e significativa (99,9%). Assim, pode-se considerar que número de *tweets* é um bom indicador de variação de vendas e pode ser usado em conjunto com as pesquisas no Google®. Entretanto, as diferentes defasagens (entre os dois medicamentos) para a maior correlação sugere que o número de *tweets* deve ser considerado com maior cuidado.

No caso da variável óbitos, para ambos os medicamentos, a melhor correlação ocorre com o uso da defasagem de um período (0,7903 e 0,8202) e confiança de 99,9%. Para a variável casos, de modo semelhante ao número de *tweets*, a melhor correlação para cloroquina ocorre sem defasagem (0,8179), enquanto, para ivermectina, ocorre com defasagem de um período (0,8607), ambos com confiança de 99,9%.

O fato de todas as correlações serem positivas parece corroborar a intuição de relacionamento das variáveis com as vendas; ou seja, quando qualquer um deles cresce, as vendas também aumentam.

Do ponto de vista de tomada de decisões da cadeia de suprimentos de medicamentos, é importante notar que as variáveis pesquisa no Google® e número de *tweets* proporcionam a avaliação em tempo real da situação. Isso possibilita antever a demanda e agilizar a tomada de decisões^30^ na gestão epidemiológica da doença. As variáveis tradicionalmente utilizadas para esse fim (casos e óbitos) são reportadas posteriormente à sua ocorrência^31^, o que dificulta seu uso de forma proativa.

A ferramenta *Google Trends*, que permite obter o número de pesquisas no Google®, é de fácil utilização, bastando digitar o termo de busca, o período desejado e a região (ou localização) de interesse. Para a obtenção do número de *tweets*, há necessidade de preparar programas de computador específicos.

Assim, o monitoramento das pesquisas no Google® e número de *tweets* permitiria prever potenciais picos de demanda para medicamentos sem eficácia para uma futura pandemia, o que permitiria tomar ações que evitassem faltas e desabastecimento para usuários reais desses medicamentos, como ocorreu com a cloroquina. Tais ações poderiam ser a exigência temporária de receitas médicas, a remoção do medicamento de plataformas de vendas pela internet ou a limitação da quantidade vendida por cliente.

Publicações acadêmicas mostraram que o comportamento de pessoas de destaque que, por meio de mídias sociais ou convencionais, promoveram o uso de medicamentos ineficazes contra a covid-19^13^ provocou um aumento nas buscas por cloroquina nos EUA quando comparado a outros países da Europa, da Oceania^9^ ou ao Canadá^8^. Isso resultou em um aumento nas vendas desses produtos no local^12^. No Brasil, declarações e postagens constantes do então presidente da república sobre os medicamentos geraram desinformação e alavancaram as buscas por tais medicamentos^1,32^.

Como sugestão de processo, agentes de saúde pública poderiam adotar as seguintes medidas em situações semelhantes de pandemias : (1) identificar figuras importantes para a disseminação de informações em mídias convencionais e sociais; (2) monitorar o número de mensagens relacionadas ao tema nas mídias sociais, considerando tanto o total de postagens quanto as postagens pelas figuras identificadas no item 1; (3) monitorar o número de buscas relacionadas ao tema realizadas em ferramentas de busca relevantes; e (4) utilizar os métodos aplicados neste trabalho (correlação de Spearman, correlação cruzada com múltiplas defasagens, análise exploratória e autocorrelação) para comparar a evolução da doença com outras séries temporais (como vendas de medicamentos, casos, óbitos, número de buscas realizadas, número de mensagens).

Com a adoção dessas medidas, seria possível identificar: (1) o potencial aumento de demanda, em tempo real; (2) a potencial ocorrência de compras em pânico em seu início, permitindo um melhor abastecimento da cadeia de suprimentos; e (3) novas tendências com relação às demandas pelos medicamentos em questão. Em conjunto, esses pontos podem auxiliar no planejamento e na escolha de estratégias dos agentes gestores da cadeia de suprimento para mitigar a falta de medicamentos, um ponto essencial para cidadãos que precisam utilizar efetivamente os remédios em questão.

## CONCLUSÕES

A cloroquina e a ivermectina foram dois medicamentos indicados no início da pandemia como suposto tratamento precoce para a covid-19, embora não houvesse estudos científicos que comprovassem sua eficácia. Pela influência de redes sociais, mídia convencional e agentes governamentais^2,4^, muitas pessoas de diversos países no mundo recorreram a esses medicamentos.

Este artigo analisou a existência de correlação entre o volume de vendas mensais no varejo desses medicamentos no Brasil com as seguintes variáveis: pesquisas no Google®, número de *tweets* relacionados a esses medicamentos, casos e óbitos registrados por covid-19 durante os dois primeiros anos da pandemia no Brasil.

Como principal contribuição deste trabalho, observou-se a existência de alta correlação para os pares de variáveis analisados. Em momentos de crises, como observado durante a pandemia, elas mostraram que são capazes de auxiliar na previsão de vendas, em especial o Google® e os *tweets,* que proporcionam uma análise em tempo real da situação. Em outras variáveis (casos e óbitos), essa correlação se mostrou mais acentuada, ao considerar uma defasagem temporal na variável de vendas, o que as tornam menos convenientes para um trabalho de previsão.

Esses resultados apontam também para uma maior necessidade do desenvolvimento de ações de coordenação de crises nas mídias sociais, devido à sua relação com o comportamento do consumidor. Acompanhar as redes sociais e a evolução de pesquisas em sites de busca proporciona uma melhor previsão de potenciais picos de demanda, permitindo um melhor planejamento de abastecimento dos agentes das cadeias de suprimentos. Ressalta-se a importância de considerar o impacto de mídias tradicionais e sociais no comportamento dos consumidores, principalmente em situações de crise.

As principais limitações deste trabalho incluem: (1) o período de análise restrito a 24 meses, inviabilizando a análise de sazonalidade de maior período; e (2) o fato de que não foi analisado o conteúdo e o sentimento dos *tweets.* O conjunto de dados gerado contém tanto *tweets* favoráveis quanto desfavoráveis ao uso desses medicamentos. Assim, estudos futuros deverão considerar a análise de sentimentos e de conteúdo dos *tweets*, para verificar se é possível melhorar modelos de previsão de vendas.
